# Modeling honey yield, defensive and swarming behaviors of Italian honey bees (*Apis mellifera ligustica*) using linear-threshold approaches

**DOI:** 10.1186/s12863-019-0776-2

**Published:** 2019-10-21

**Authors:** Sreten Andonov, Cecilia Costa, Aleksandar Uzunov, Patrizia Bergomi, Daniela Lourenco, Ignacy Misztal

**Affiliations:** 1 Department of Animal Breding and Genetic, Swedish University of Animal Sceinces, P.O. Box 7023, 75007 Uppsala, Sweden; 2 Faculty of Agricultural Sciences and Food, Ss. Cyril and Methodius University, P.O. Box 297, 1000 Skopje, Macedonia; 3 CREA Research Centre for Agriculture and Environment, Via di Saliceto 80, 40128 Bologna, Italy; 4 Landesbetrieb Landwirtschaft Hessen, Bee Institute, Erlenstrasse 9, 35274 Kirchhain, Germany; 5 Department of Animal and Dairy Science, University of Georgia, Athens, GA 30602 USA

**Keywords:** Threshold model, Heritability, Genetic correlation, Predictability, Genetic evaluation

## Abstract

**Background:**

Genetic improvement of honey bees is more difficult compared to other livestock, due to the very different reproductive behavior. Estimation of breeding values requires specific adjustment and the use of sires in the pedigree is only possible when mating of queens and drones is strictly controlled. In the breeding program of the National Registry for Italian Queen Breeders and Bee Producers the paternal contribution is mostly unknown. As stronger modeling may compensate for the lack of pedigree information, we tested two models that differed in the way the direct and maternal effects were considered. The two models were tested using 4003 records for honey yield, defensive and swarming behaviors of Italian honey bee queens produced between 2002 and 2014. The first model accounted for the direct genetic effect of worker bees and the genetic maternal effect of the queen, whereas model 2 considered the direct genetic effect of the queen without maternal effect. The analyses were performed by linear (honey production) and threshold (defensive and swarming behavior) single-trait models; estimated genetic correlations among traits were obtained by a three-trait linear-threshold model.

**Results:**

For all traits, the highest predictability (correlation between breeding values estimated with and without performance records) was obtained with model 2, where direct genetic effect of queens was considered. With this model, heritability estimates were 0.26 for honey yield, 0.36 for defensive behavior, and 0.34 for swarming behavior. Multi-trait estimation resulted in similar or higher heritability estimates for all traits. A low, positive genetic correlation (0.19) was found between honey yield and defensive behavior, whereas the genetic correlation between honey yield and swarming behavior was moderate (0.41). A strong, positive genetic correlation was found between defensive and swarming behaviors (0.62). Predictability for multi-trait evaluations was higher for honey yield (0.46) and defensive behavior (0.30) but almost identical for swarming behavior (0.45) compared to corresponding single-trait predictability.

**Conclusions:**

Multi-trait evaluation using a model that accounts for the direct genetic effect of queen was the best approach for breeding value estimation of Italian honey bees. The results suggest a new direction for selection of linear and categorical traits in breeding programs where drone origin is unknown.

## Background

Honey bees (*Apis mellifera)* represent a biologically and economically valuable species. They contribute to pollination of wild plants and crops and provide direct income to beekeepers who collect honey and other hive products from managed colonies. Programs for genetic improvement of honey bees have been developed and implemented [1–3], but progress has not been as successful as in other species. In comparison with other livestock that have been the subject of genetic improvement, honey bee colonies are made up of thousands of individuals belonging to different castes and generations and are interdependent on one another to guarantee functioning of the super-organism [4]. Honey bees thus have a different biology and mating system compared to other livestock: a virgin queen is mated with 10 to 20 drones once in her lifetime, and the sperm is stored in the spermatheca to fertilize eggs during her reproductive life [5–8]; after mating, a queen lays fertilized eggs that develop into workers (or new queens, if necessary) and unfertilized eggs that develop into drones. Drones are haploid copies of their queen mother and represent only the queen’s genetic contribution and not any of her mates [9]. The mating between a virgin queen and 10 to 20 drones takes place in mid-air, several kilometers distant from the hives of origin. The places where the mating happens are called drone congregation areas (DCA), where all drones (of known and unknown origin) from the area are contributing.

In breeding programs, mating can be controlled by the use of isolated mating stations (in which only colonies of known origin are present usually within a radius of 3 to 10 km) or instrumental insemination.

Traditional traits of interest in apiculture are yields (such as honey production) and behaviors (such as defensiveness, calmness, and swarming), for which routine collection of data exists in many countries [10]. More recently, traits involved in resistance to parasites (such as *Varroa destructor*) and to fungal and bacterial diseases (such as *Nosema* spp. and Foulbrood) are being considered in breeding programs [2, 3, 11].

Because honey bees are social insects, the performance of the honey bee colony is the joint performance of the queen and of her daughters, the worker bees. The queen contributes with her egg-laying capacity, which maintains the number of workers necessary for performing colony activities as well as homeostasis within the hive; she also regulates the functions of the colony by means of pheromone production. The genetic makeup of the worker bees may affect the way they react to the queens’ regulatory activity as well as directly affecting foraging, defensive, and swarming behaviors.

The challenge in honey bee breeding is that evaluations are based on performance of the colony, which includes both queen and worker effects; however, for reproduction of future generations, only the queen is used. Because of these peculiarities, the methods used in honey bee breeding programs are modifications of those currently used in livestock breeding. The overall breeding goal is to produce virgin queens from superior colonies, which can then be mated to drones also from superior colonies. To take account of the haplo-diploid reproduction system, groups of sister queens (daughters of the superior colony) should be used for drone production [12].

Genetic evaluations rely on the relationships among individuals and the ability to separate genetic from non-genetic effects for traits of interest. In the honey bee colony, the two main sources of genetic influence are the queen and the workers. Bienefield and Pirchner [13] developed a method in which the genetic effects of workers and the queen were included in the evaluation. This approach was used in several studies in which the breeding program used drones produced by sister queens [14–16]. For some other genetic evaluations, only the direct genetic effect of the queens was considered [11, 17–20]. For populations with limited genetic ties among colonies and lack of sufficient information on drone origin, the maternal effect of queens could not be successfully estimated [11, 18].

In livestock, threshold models have been introduced to improve genetic analyses of categorical traits [21, 22]. The predictability of breeding values from a threshold animal model is higher than from an equivalent linear animal model for discrete traits [23, 24]. Threshold methodology has also been extended to multi-trait analyses that consider one or more continuous correlated traits and unequal design [25].

Because drone origin in some honey bee breeding programs is not known due to lack of mating control, the aim of this study was to estimate breeding values using performance testing data with only maternal information. Such a situation occurs in Italy, for most breeders belonging to the National Registry for Italian Queen Breeders and Bee Producers, where measurements are made on a breeding population in which mating is not strictly controlled. In addition, studies have shown that genotype-by-environment interactions affect colony performance [26, 27] and that environmental effects sometimes mask differences among genotypes [28]. Thus, the specific objectives of this study were to (1) estimate genetic parameters for honey yield and defensive and swarming behaviors by linear or threshold approaches; and (2) estimate genetic correlations among honey yield and defensive and swarming behaviors using a linear-threshold approach.

## Results

Single-trait estimates of variance components and heritabilities for Models 1 and 2 and their predictabilities are summarized in Table 1 for HY, DB, and SB. Multi-trait heritability estimates, genetic correlations, and predictabilities are in Table 2.

**Table 1:** Variance component estimates and genetic correlations for honey yield and defensive and swarming behaviors

Statistic	Honey yield (kg)		Defensive behavior (1–5)		Swarming behavior (1–5)	
	Model 1	Model 2	Model 1	Model 2	Model 1	Model 2
${\sigma}_W^2$	71.12 (1.94)	–	12.93 (3.42)	–	17.46 (4.04)	–
${\sigma}_{MQ}^2$	24.73 (2.13)	–	5.97 (2.14)	–	8.81 (1.80)	–
*Cov*_*W-MQ*_	0.34 (2.56)	–	−8.38 (1.42)	–	−11.39 (2.60)	–
${\sigma}_Q^2$	–	50.14 (7.15)	–	2.59 (0.57)	–	4.18 (0.92)
${\sigma}_{py- ta}^2$	110.75 (14.87)	105.85 (14.91)	1.76 (0.53)	1.33 (0.42)	1.83 (0.53)	1.64 (0.48)
${\sigma}_e^2$	6.97 (0.60)	35.98 (5.49)	0.24 (0.28)	3.24 (1.47)	0.43 (0.50)	6.35 (1.85)
${\sigma}_p^2$	213.91	191.97	12.52	7.17	39.92	12.17
${h}_W^2$	0.33 (0.03)	–	> 1 (0.08)	–	0.44 (0.08)	–
${h}_{MQ}^2$	0.12 (0.01)	–	0.48 (0.12)	–	0.22 (0.13)	–
${h}_C^2$	0.45 (0.02)	–	0.17 (0.08)	–	0.20 (0.08)	–
*r*_*W* − *MQ*_	0.01 (0.01)	–	−0.95 (0.12)	–	−0.92 (0.13)	–
${h}_Q^2$	–	0.26 (0.04)	–	0.36 (0.01)	–	0.34 (0.01)
*r*_*EBV-Y*_	0.42	0.43	0.18	0.27	0.19	0.46

**Table 2:** Correlations, heritability, and predictability from a multi-trait threshold linear model for Italian honey bee traits

Trait	Honey yield	Defensive behavior	Swarming behavior
Honey yield	0.25^1^ ± 0.04	0.19^NS^ ± 0.12	0.41^**^ ± 0.14
Defensive behavior		0.43 ± 0.05	0.62^**^ ± 0.11
Swarming behavior			0.42 ± 0.05
Predictability	0.46	0.30	0.45

NS = *P* > 0.05; ** *P* < 0.01

^1^Genetic correlations (± standard errors) are above the diagonal, and heritability estimates (± standard errors) are on the diagonal. Predictability was the correlation between breeding values estimated with and without performance information

In the honey yield estimates with Model 1, the genetic components were the genetic direct effects of worker bees, the genetic maternal effect of the queen, and their covariance. Heritability of the worker direct effect was more than double the heritability of the queen maternal effect (0.31 ± 0.03 and 0.12 ± 0.01, respectively), whereas heritability at the colony level was 0.42 ± 0.02. Genetic correlation estimation between worker bees and queen maternal effect was zero. In addition, the residual variance was low, accounting for about 3% of the phenotypic variance. Predictability of model 1 for HY was 0.42. Model 2 was simpler than Model 1. Estimated heritability with Model 2 for HY was moderate (0.26 ± 0.04), residual variance accounted for about 18%, and predictability was similar to the one achieved with Model 1. In both models the random non-genetic component (random effect of interaction between performance test year and tester apiary) accounted for slightly more than half of the phenotypic variance.

Estimates of DB with Model 1 were unsuccessful, resulting in heritabilities for worker effect over 1, although the queen maternal effect and colony genetic effects were 0.48 ± 0.12 and 0.17 ± 0.08. A strong, negative genetic correlation (− 0.95 ± 0.12) was estimated between worker and queen maternal effects. In addition, the standard error of the residual variance was higher than the variance itself. When applying the less complex model with only the queen genetic effect (Model 2), the estimated heritability was 0.36 ± 0.01. Predictabilities of Model 1 and Model 2 for DB were 0.18 and 0.27.

Estimated variance components include additive genetic variances for worker bee (${\sigma}_W^2$), queens maternal (${\sigma}_{MQ}^2$), covariance between worker bees and queens maternal effect (*Cov*_*W-MQ*_) in Model (1); genetic direct effect of queen (${\sigma}_Q^2$) in Model (2), random effect of interaction between performance test year and tester apiary variance (${\sigma}_{py- ta}^2$) residual variances (${\sigma}_e^2$) and phenotypic variance (${\sigma}_p^2$) for both models. Heritabilities in Model 1 were estimated for genetic direct effect of worker bee (${h}_W^2$), queens’ genetic maternal (${h}_{MQ}^2\Big)$, and colony (${h}_C^2$), as well as genetic correlations between direct genetic effect of worker bees and queens’ genetic maternal (*r*_W-MQ_). Heritability for genetic direct queen effect (${h}_Q^2$) was obtained with Model 2. Approximate standard errors are given in brackets. *r*_*EBV-Y*_ is predictability of both models as correlation between EBVs estimated with complete information and EBVs estimated without performance (i.e., using only pedigree information).

Similar to DB, in the evaluation of SB with Model 1 the estimated standard error of the residual variance was above the estimated residual variance. For SB, heritabilities of 0.44 ± 0.08, 0.22 ± 0.13 and 0.20 ± 0.08 were estimated for genetic direct effect of the worker, genetic maternal effect of the queen, and the colony, respectively. Also, the genetic correlation between worker effect and queen maternal was − 0.92 ± 0.13. Estimated heritability of genetic direct effect of queen for this trait was 0.34 ± 0.01 (Model 2). A better predictability for SB was obtained with Model 2 than with Model 1 (0.46 vs 0.19).

In the analysis using Model 2 for all three traits together (Table 2), the estimated heritability for HY was almost identical to the one obtained in the single-trait evaluation. The heritabilities estimated with the three-trait model for both DB and SB were about 0.42, therefore, greater than those obtained with single-trait models (around 0.35). Genetic correlation between HY and DB was weak (0.19 ± 012) and moderate between HY and SB (0.41 ± 0.14). Genetic correlations between behavioral traits (DB and SB) was positive (0.62 ± 011). However, the estimated genetic correlation between HY and DB was not significantly different form zero. Other estimated correlations were significantly different form zero. The three-trait predictability was higher for HY (0.46) and DB (0.30) but almost identical for SB (0.45) compared to predictabilities from corresponding single-trait evaluation.

## Discussion

The present study investigated alternative models to estimate genetic parameters and breeding values for traits of apicultural interest in Italian honey bees (*A. m. ligustica*). It used phenotypes from performance tests carried out within a national breeding program. Two concepts of genetic effects were applied in order to reach better model predictability. In Model 1 we accounted for genetic direct effect of worker bees and genetic maternal effect of the queen, whereas in Model 2 only the genetic direct effect of the queen was used. Unless all drones that mate with a particular queen are known, distinguishing between the contributions of queens and workers to colony performance is difficult. The size of the sub-family of workers, offspring of a single drone, and its presence within the colony at the time the behavioral traits were measured also affect the different contributions.

Model 1 allowed separation of variance components (additive genetic, environmental, and residual). However, the genetic correlation between the genetic direct effect of worker bees and genetic maternal effect of the queen was nearly zero for HY (0.01) and strongly negative for behavior traits (− 0.95 for DB and − 0.92 for SB). Applying Model 1 resulted in low residual variance, particularly in categorical traits (DB and SB), where the standard error was bigger than the value itself. In addition, the genetic direct effect of worker bees for DB was overestimated (over 1), suggesting poor fit of the model. Two studies [13, 29] found a strong negative genetic correlation between the worker effect and queen maternal effects for honey production; for behavior traits, our correlations were similar to the values reported in the two studies. Worker bees inherit about 50% of genes and 100% of maternal effect from the queen. To evaluate maternal effect, queen performance is of particular importance. This information can be accessed through pedigree ties where siblings, parents, and ancestors are known and the pedigree is deep. If the origin of the queen is known for fewer than three generations as in the considered Italian data, direct genetic effect of worker and genetic maternal effect of the queen cannot be estimated successfully. Zakour et al. [18] also reported that complex models with genetic maternal queen effect could not be applied to Syrian honey bee data.

For all traits, predictability was greatest with a queen direct genetic effect in the model, as in Model 2. For HY, predictabilities were very similar in Models 1 and 2 (Table 2). However, for behavior traits, predictability was higher when a genetic direct effect of the queen was considered instead of genetic direct effect of the worker and genetic maternal effect of the queen (0.09 higher for DB and 0.27 higher for SB). Therefore, we recommend a model that includes direct genetic effect of queens for current evaluation of honey bee performance in Italy.

Heritability estimates from Model 2 were 0.26 for HY, 0.36 for DB, and 0.34 for SB. Zakour et al. [18] and Brascamp et al. [29] estimated a similar moderate heritability of 0.27 for honey production using different methods and models. However, Padilha et al. [19] and Najafgholian et al. [17] reported a lower heritability of 0.17 and 0.18, respectively. Tahmasbi et al. [20] exploring only genetic direct effect of queens, reported heritability of honey yield in Iranian honey bee of 0.22. Although our defensive behavior data were scored using a larger scale than in other studies, the moderate heritability estimate of 0.36 was close to the gentleness estimate of Brascamp et al. [29] but greater than the 0.08 (gentleness) reported by Zakour et al. [18]. Najafgholian et al. [17] estimated heritability for defense behavior as 0.44, which is greater than the results from in study. Our estimate of heritability for swarming behavior was greater than the estimates reported by Brascamp et al. [29], but identical to estimates of Najafgholian et al. [17]. In our study, defensive and swarming behaviors were treated as categorical traits and evaluated with threshold models. Advantages of threshold over linear models have been demonstrated in genetic evaluation of livestock [23, 24]. Our estimates may be more reliable than the ones found in other studies because of the methodology used and model robustness.

### Multi-trait model

Heritability for HY using the multi-trait model was almost identical to the ones in the single-trait models, whereas heritabilities for DB and SB were greater than in the single-trait models. Multi-trait models are usually more accurate than single-trait models because they include additional information and relationships among traits [30].

The genetic correlation between HY and DB was weak (0.19) but moderate and positive between HY and SB (0.41). A strong positive genetic correlation (0.62) was found between DB and SB. Because the standard error for the genetic correlation between HY and DB was large relative to the estimated correlation, the correlation was probably not significantly different from zero. Standard errors relative to estimated correlations were considerably smaller for correlations between HY and SB and between DB and SB, which suggests that the estimates are reliable. Unfortunately, information on genetic correlations between honey bee traits is limited. In addition, comparisons are difficult because methodology and trait definition varies among studies. Bienefeld and Pirchner [31] estimated a moderate positive genetic correlation (0.31 ± 0.55) between honey production and aggressiveness. Zakour et al. [18] estimated a moderate negative genetic correlation (− 0.50 ± 0.93) between honey production and gentleness, whereas Brascamp et al. [29] found almost no genetic correlation (− 0.07 ± 0.16). The latter also estimated a very strong negative genetic correlation (− 0.82 ± 0.30) between honey production and swarming behavior and a strong positive genetic correlation (0.65 ± 0.29) between gentleness and swarming behavior. Approximate standard errors in all those studies were large, which suggests that the estimated correlations were not significant.

## Conclusions

An animal model that included fixed effects for performance test year and tester apiary as well as a random effect for their interaction, together with genetic direct effect of the queen is the most accurate for genetic evaluation of honey yield and defensive and swarming behaviors in honey bees in Italy. Stable variance components and highest predictive ability for all traits are possible when genetic direct effect of worker and genetic maternal effect of the queen are excluded and only the queen direct genetic effect is used in the model. Estimates of heritability and predictability are improved with a multi-trait model that includes honey yield and defensive and swarming behaviors. This study reports the first estimation of genetic parameters in Italian honey bees, and the results may represent a possible direction for the genetic selection of linear and categorical traits in honey bees.

## Methods

### Data

Results of performance testing from *Apis mellifera ligustica* colonies were obtained from the National Registry for Italian Queen Breeders and Bee Producers, which is supported by the Italian Ministry of Agriculture for the improvement and conservation of the autochthonous Italian honey bee subspecies. The member breeders are responsible for queen production and performance testing, whereas a governmental research center (CREA-AA, previously known as the Honey Bee Research Unit) is responsible for anonymous queen distribution to performance testing centers, where expert beekeepers perform blind evaluations of colony performances. The CREA-AA is also in charge of data collection and upload of validated data to the www.beebreed.eu platform to be processed with a modified best linear unbiased prediction animal model for estimation of breeding values [14].

The evaluation program starts each year in June–July with production of sister queens groups (10–15 per group). These are partly kept by the producing breeders and partly sent for anonymous evaluation. Queens are assigned to the performance testing centers so that different lines are distributed across apiaries, with a bias towards the area of origin. Standardization within and between test apiaries (such as equalization of initial colony strength and infestation level, prevention of bee drifting behavior, and unified management techniques) is a prerequisite for unbiased and proper evaluation of the test colonies [2]. Evaluation of the queens starts in March–April of the following year and lasts until October [32]. Three traits of apicultural interest were consistently measured: honey yield (HY), defensive behavior (DB), and swarming behavior (SB). Most internationally recognized testing protocols assess the behavioral traits with a 4-score range [2, 3]. However, the Italian national breeding program uses 5-score range for assessment of gentleness, swarming, and spring development.

The dataset included 4003 records for HY and DB and SB for queens born from 2002 through 2014. Testing was performed in 31 apiaries located in nine regions in Italy. After data verification, 3974 colonies with records remained. Each record consisted of a unique identification code for the tested colony’s queen and her dam (based on region of origin, breeder, an integer number, and birth year), the tester, the testing apiary, and measurements of HY (kilograms), DB and SB (scored 1–5). As mentioned above, in the Italian Registry honey bee breeding program, mating is not strictly controlled, and most breeders use several paternal lines at the same time. Therefore, information about the queens’ mates was not included. Phenotypic data are described in Table 3.

**Table 3:** Descriptive statistics for honey yield, defensive behavior, and swarming behavior of Italian honey bee colonies

Trait	N^a^	Min.	Max.	Mean	SD
Honey yield (kg)	3974	1	135	22.64	18.25
Defensive behavior (scores 1–5)	3931	1	5	3.90	0.69
Swarming behavior (scores 1–5)	3865	1	5	3.87	0.93

^a^Data included records for queens born from 2002 through 2014

**Fig. 1:**
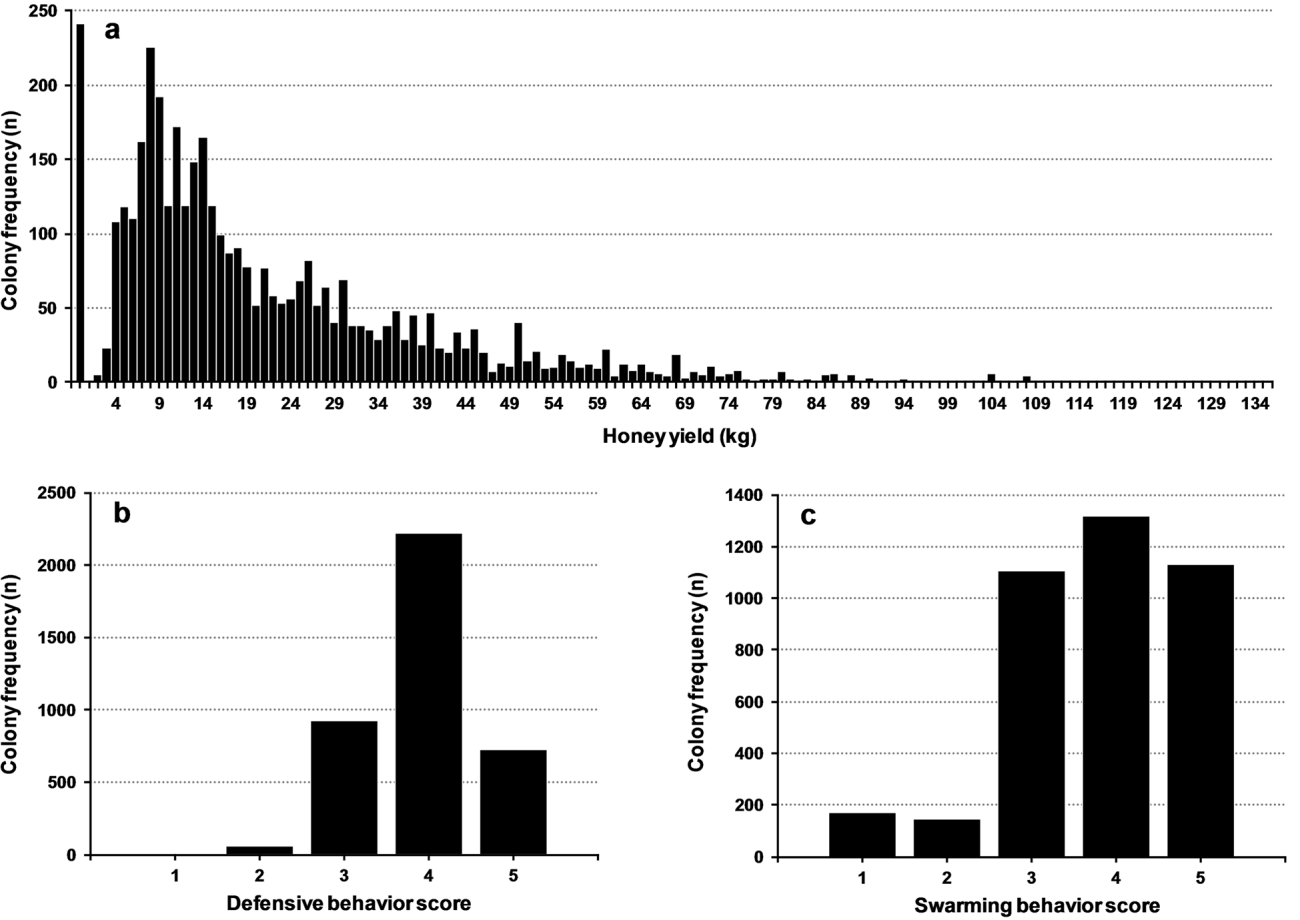
Distribution of colonies’ performance for kg honey yield (**a**), scores of defensive (**b**) and swarming behaviors (**c**) in Italian honey bee

Honey yield was recorded as the weight difference of combs before and after extraction of honey, and the final yield is a sum of all honey harvested from a colony over a season. Of the total 3974 tested colonies, 241 had 0 kg of honey yield (i.e. failed to produce any honey). For the remaining 3733 colonies, honey yield ranged from 1 to 135 kg (Fig. 1a). Almost 50% of colonies with positive honey yield ranged between 4 and 15 kg. However, all 3974 records for HY were used in the analysis.

Defensive behavior and SB had less records compared to HY (Table 3). Both DB and SB were assessed on a scale from 1 to 5 during at least three specific colony inspections during the testing period [33]. Defensive behavior is the bees’ response and reaction during colony inspection; low scores indicate strong defensive behavior (bees stinging and following the intruder), and high scores indicate that the colony is gentle (no stinging incidents), which is desirable for most beekeepers. The average score rounded to one decimal was used as the overall measure of the colony’s defensive behavior. Swarming behavior is the tendency of the colony to reproduce itself by splitting, which in modern beekeeping is considered to be a negative event because it causes loss of bees for honey production. A higher score for swarming behavior indicates a lower swarming tendency. The average score rounded to one decimal was used as the overall measure of the colony’s swarming behavior, unless the colony actually swarmed; in that case, the lowest score was assigned [33]. Most of the records for behavioral traits (82.7% for defensive and 88.5% for swarming) were scored as integers; therefore, decimal scores were grouped towards closest integer score. After grouping, 55.4% of colonies were scored 4 for DB (Fig. 1b); for SB, the majority of colonies (88.7%) were scored 3 to 5 (Fig. 1c). A total of 3650 colonies had records for all three traits (HY, DB and SB).

The genealogical data contained unique identification numbers for all queens, and their dams were traced back as far as possible. The complete pedigree data included 4160 queens. Among those, 186 queens were defined as base population, and 1625 were dam queens with an average family size of 2 (ranging from 1 to 33). Sixty-four groups of relatives had more than 4 members. The pedigree included 365 grand dam queens with an average family size of 13 (ranging from 1 to 52). Because the breeding scheme is open, new entries of queens with unknown origin were present, but such cases have been minimal during the last few years. Two pedigree files were prepared. The first pedigree file (*n* = 4160) contained the unique identification number of queen, unknown sire, unique identification number of dam and birth year of that particular queen. The second pedigree file was created in two steps in order to evaluate the effect of worker bees and queen within colonies. It contained the information for two types of individuals, e.g., worker bees as a group of individuals in the colonies and queens. First a unique identification number for the worker bees within each colony was generated (*n* = 3974) followed by unknown sire, unique identification number of queen (dam of the worker bees) and test year of that particular colony. Then, the information for the queens including their ancestors and year of birth was added (identically to first pedigree file; *n* = 4160). This procedure resulted in pedigree file with a total of 8134 lines (i.e., 3974 lines for worker bees and 4160 lines for queens).

### Statistical models

In honey bees the performance is measured at the colony level, to which contribute both the worker bees and their dam – the queen. Workers contribute to honey yield by their flying and harvesting abilities, whereas the queen has the responsibility to: a) maintain sufficient number of worker bees within colony through her egg laying capacity and b) regulate and stimulate worker activity by producing pheromones [33]. In honey bee breeding programs the aim is to produce superior queens who will produce superior offspring in order to improve the performance of colonies. In practice, the breeding value of the queen is the sum of the breeding value of the queen itself and of her progeny of worker bees. Before consideration of the model in use, several options were tested for non-genetic effects. The final statistical Model 1 contained the direct genetic effect of worker bees and genetic maternal effect of queen:

1 $$ {Y}_{ijkl}={py}_i+{ta}_j+ py-{ta}_k+{w}_l+{mq}_l+{e}_{ijkl} $$

where: *y*_*ijkl*_ = HY, DB or SB for colony *l* within performance year *i* in tester apiary *j* and interaction *k*; *py*_*i*_ = fixed effect of performance test year *i* (*i* = 1 to 13); *ta*_*j*_ = fixed effect of tester apiary *j* (*j* = 1 to 72); *py-ta*_*k*_ = random effect of interaction between performance test year and tester apiary (*k* = 1 to 207); *w*_*l*_ = random genetic direct effect of worker bees in colony *l*; *mq*_*l*_ = random genetic maternal effect of queen of colony *l*; *e*_*ijkl*_ = residual.

Alternatively, the genetic direct effect of queens can be also estimated, assuming the queen is capturing the worker bees’ effect within the colony. Thus, in Model 2 all non-genetic effects are identical to the ones in Model 1, but the genetic direct effect is limited to the random effect of queen in colony *l* (*q*_*l*_).

2 $$ {Y}_{ijkl}={py}_i+{ta}_j+ py-{ta}_k+{q}_l+{e}_{ijkl} $$

In the beginning, both models were tested for each trait separately (i.e., single-trait model). The best performing model was used in a three-trait analysis.

Based on Model 1, we estimated direct heritability for workers and maternal heritability for queens. Additionally, total genetic variance on colony level was taken as a sum of variance of direct effect of worker bees, maternal effect of queen and twice the covariance between them. It can be expressed as ${\sigma}_C^2={\sigma}_W^2+{\sigma}_{MQ}^2+2{Cov}_{W- MQ}$. The total genetic variance on colony was used for calculation of heritability for colony. Based on Model 2 we calculated direct heritability for queens, only.

### Model comparison

Models were compared for their fit and ability to predict future performance. For variance component estimates and heritabilities, standard errors were calculated. For ability to predict future performance, colony performance for 150 queens that had families with more than three members and known grandparents were removed from the dataset (reduced data). Estimated breeding values (EBV) were obtained using the reduced dataset for each tested model. Pearson correlation between EBV in the reduced data (*r*EBV) and phenotypes (*Y*) that were removed was used as a measure of predictive ability for each model. The strongest correlation points to a better model. The expected value of this correlation [*E*(*r*)] is as follows: *E*(*r*) = *k* × *rEBV-BV*, where *k* is a constant, being the ratio between standard deviation of true breeding values and that of phenotype [e.g., maximum square root of heritability (h^2^), when no fixed effects affect phenotypic variance]; and *rEBV-BV* is the correlation between true breeding value of the animal and the predicted (pedigree) breeding value.

### Analyses

Honey yield was considered to be a continuous trait, whereas defensive and swarming behaviors were considered categorical traits with 5 classes. Because the origin of drones was not known in the considered breeding population, in preliminary analyses we introduced their influence using phantom drones or genetic groups in the pedigree file. These methods should allow more genetic ties between queens and colonies, but none of them improved estimation of genetic parameters and thus were not used. The AIREMLF90 software [34] was used to estimate variance components of single-trait linear models for honey yield with a convergence criterion of 10^− 12^. The THRGIBBS1F90 program [35] was used to estimate variance components of single-trait threshold and all multi-trait linear-threshold models with 100,000 Gibbs sampling iterations for single-trait and 500,000 for multi-trait evaluations. A burn-in of 10% of the initial iterations was used. The POSTGIBBSF90 program [35] was used to check convergence and calculate posterior means. The EBV were computed using BLUPF90 [34] and a convergence criterion of 10^− 12^ for linear traits and THRGIBBS1F90, with an option to store solutions assuming variance components are known, for threshold traits.

## Abbreviations

[*E*(*r*)]: Expected value of this correlation [*E*(*r*)]
CREA-AA: Council for Agricultural Research and Economics
DB: Defensive Behavior
DCA: Drone Congregation Area
EBV: Estimated Breeding Value
h^2^: Heritability
HY: Honey Yield
*r*EBV: Pearson correlation between EBV in the reduced data and phenotypes (*Y*)
*rEBV-BV*: Pearson correlation between true breeding value of the animal and the predicted (pedigree) breeding value.
SB: Swarming Behavior

## References

[1] Rinderer TE (1986). Bee genetics and breeding.

[2] Büchler R, Andonov S, Bienefeld K, Costa C, Hatjina F, Kezic N (2013). Standard methods for rearing and selection of *Apis mellifera* queens. J Apic Res.

[3] Tiesler FK, Bienefeld K, Büchler R (2016). Selektion bei der Honigbiene.

[4] Moritz RFA, Fuchs S (1998). Organization of honeybee colonies: characteristics and consequences of a superorganism concept. Apidologie.

[5] Winston ML (1987). The biology of the honey bee.

[6] Schlüns H, Moritz RFA, Neumann P, Kryger P, Koeniger G (2005). Multiple nuptial flights, sperm transfer and the evolution of extreme polyandry in honeybee queens. Anim Behav.

[7] Hernández-García R, de la Rúa P, Serrano J (2009). Mating frequency in *Apis mellifera iberiensis* queens. J Apic Res.

[8] Koeniger G, Koeniger N, Ellis J, Connor L (2014). Mating biology of honey bees (*Apis mellifera*).

[9] Dzierzon J (1845). Gutachten über die von Herrn Direktor Stöhr in ersten und zweiten Kapitel des General-Gutachtens aufgestellten Fragen. Bienenzeitung.

[10] Lodesani M, Costa C (2003). Bee breeding and genetics in Europe. Bee World.

[11] Boecking O, Bienefeld K, Drescher W (2000). Heritability of the Varroa-specific hygienic behaviour in honey bees (Hymenoptera: Apidae). J Anim Breed Genet.

[12] Ruttner F (1988). Breeding techniques and selection for the breeding of the honey bee.

[13] Bienefeld K, Pirchner F (1990). Heritabilities for several colony traits in the honeybee (*Apis mellifera carnica*). Apidologie.

[14] Bienefeld K, Ehrhardt K, Reinhardt F (2007). Genetic evaluation in the honey bee considering queen and worker effects – a BLUP-animal model approach. Apidologie.

[15] Ehrhardt K, Büchler R, Bienefeld K (2010). Genetic parameters of new traits to improve the tolerance of honeybees to varroa mites. Proceedings of 9th world congress on genetics applied to livestock production.

[16] Gupta P, Reinsch N, Spötter A, Conrad T, Bienefeld K (2013). Accuracy of the unified approach in maternally influenced traits – illustrated by a simulation study in the honey bee (*Apis mellifera*). BMC Genet.

[17] Najafgholian J, Pakdel A, Thahmasbi G, Nehzati G (2011). New approach for estimating of heritability in honeybee population. Int J Plant Anim Env Sci.

[18] Zakour MK, Ehrhardt K, Bienefeld K (2012). First estimate of genetic parameters for the Syrian honey bee *Apis mellifera syriaca*. Apidologie.

[19] Padilha AH, Sattler A, Cobuci JC, McManus CM (2013). Genetic parameters for five traits in Africanized honeybees using Bayesian inference. Genet Mol Biol.

[20] Tahmasbi G, Kamali MA, Ebadi R, Nejati Javaremi A, Babaei M, Gharadaghi AA (2015). Genetic trends and parameters of honey production, swarming and defense behavior in Iranian honey bee (*Apis mellifera meda*) colonies. J Agric Sci Technol.

[21] Gianola D, Foulley JL (1983). Sire evaluation for ordered categorical data with a threshold model. Genet Sel Evol.

[22] Gilmour AR, Anderson RD, Rae AL (1985). The analysis of binomial data by a generalized linear mixed model. Biometrika.

[23] Varona L, Misztal I, Bertrand JK (1999). Threshold-linear versus linear-linear analysis of birth weight and calving ease using an animal model. I. Variance component estimation. J Anim Sci.

[24] Ramirez-Valverde R, Misztal I, Bertrand JK (2001). Comparison of threshold vs linear and animal vs sire models for predicting direct and maternal genetic effects on calving difficulty in beef cattle. J Anim Sci.

[25] Janss LLG, Foulley JL (1993). 1993. Bivariate analysis for one continuous and one threshold dichotomous trait with unequal design matrices and an application to birth-weight and calving difficulty. Livest Prod Sci.

[26] Costa C, Lodesani M, Bienefeld K (2012). Differences in colony phenotypes across different origins and locations: evidence for genotype by environment interactions in the Italian honeybee (*Apis mellifera ligustica*). Apidologie.

[27] Hatjina F, Costa C, Büchler R, Uzunov A, Drazic M, Filipi J (2014). Population dynamics of European honey bee genotypes under different environmental conditions. J Apic Res.

[28] Meixner MD, Francis RM, Gajda A, Kryger P, Andonov S, Uzunov A (2014). Occurrence of parasites and pathogens in honey bee colonies used in a European genotype-environment interactions experiment. J Apic Res.

[29] Brascamp EW, Willam A, Boigenzahn C, Bijma P, Veerkamp RF (2016). Heritabilities and genetic correlations for honey yield, gentleness, calmness and swarming behaviour in Austrian honey bees. Apidologie.

[30] VanRaden PM, Tooker ME, Wright JR, Sun C, Hutchison JL (2014). Comparison of single-trait to multi-trait national evaluations for yield, health, and fertility. J Dairy Sci.

[31] Bienefeld K, Pirchner F (1991). Genetic correlations among several colony characters in the honey bee (Hymenoptera: Apidae) taking queen and worker effects into account. Ann Entomol Soc Am.

[32] Costa C, Lodesani M, Ridolfi F, Cobey S (2015). Queen rearing and bee breeding in Italy: a brief history and overview, with focus on sourcing stock for the U.S. Am Bee J.

[33] Ministero delle Politiche Agricole Alimentari e Forestali (2013). D.M. n. 1839 del 30/1/2013 con cui viene approvato il Disciplinare attualmente vigente dell’Albo Nazionale degli Allevatori di Api Italiane e relative Norme Tecniche.

[34] Misztal I, Tsuruta S, Strabel T, Auvray B, Druet T, Lee DH (2002). BLUPF90 and related programs (BGF90). Proceeding of 7th world congress on genetics applied to livestock production.

[35] Tsuruta S, Misztal I (2006). THRGIBBSF90 for estimation of variance components with threshold and linear models. Proceeding of 8th world congress on genetics applied to livestock production.

